# Supplementary material for: Decoupling between Translational Diffusion and Viscoelasticity in Transient Networks with Controlled Network Connectivity
Source: Gels. 2022 Dec 16;8(12):830. doi: 10.3390/gels8120830 (PMC9778075; doi:10.3390/gels8120830)
Supplement: Supplementary file 1 [file gels-08-00830-s001.zip › gels-2093140-supplementary.pdf]

gels

Supplementary Materials

Decoupling between Translational Diffusion and Viscoelasticity in Transient Networks with Controlled Network Connectivity

Takuya Katashima [1,], Ryunosuke Kobayashi [1], Shohei Ishikawa [1], Mitsuru Naito [2], Kanjiro Miyata [1,2], Ung-il Chung [1,3], and Takamasa Sakai [1,4]*

1. Department of Bioengineering, School of Engineering, The University of Tokyo, 7-3-1 Hongo, Bunkyo-ku, Tokyo 113-8656, Japan
2. Department of Materials Engineering, Graduate School of Engineering, The University of Tokyo, 7-3-1 Hongo, Bunkyo-ku, Tokyo 113-8656, Japan
3. Center for Disease Biology and Integrative Medicine, Graduate School of Medicine, The University of Tokyo, 7-3-1, Hongo, Bunkyo-ku, Tokyo 113-8656, Japan
4. Department of Chemistry and Biotechnology, Graduate School of Engineering, The University of Tokyo, 7-3-1 Hongo, Bunkyo-ku, Tokyo 113-8656, Japan

** To whom correspondence should be addressed.*

E-mail: katashima@tetrapod.t.u-tokyo.ac.jp

TEL: +81-3-5841-1863

1. Comparison of viscoelasticity between Alexa-labeled and neat samples

Figure S1 shows the results of the dynamic viscoelasticity to compare the Alexa-labeled and neat Tetra-PEG slimes. Despite fluorescence modification, the viscoelastic spectra were well overlapped, suggesting that the effect of the end modification is negligible for viscoelasticity.

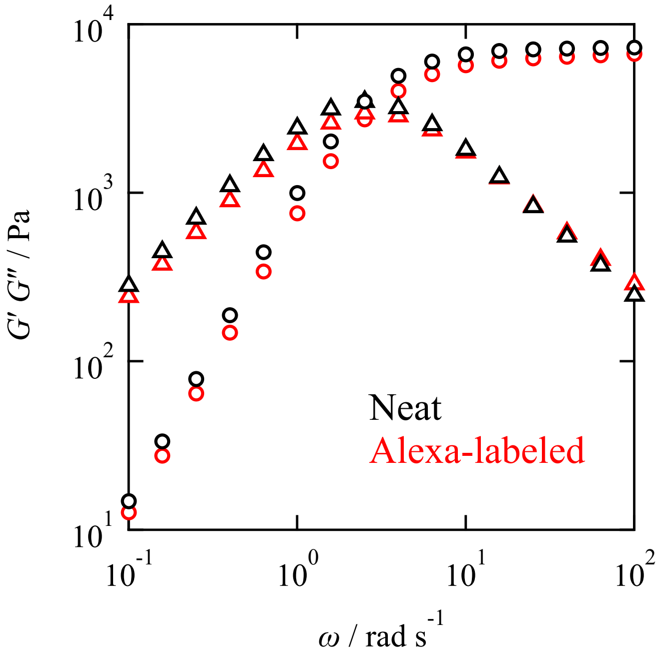

Figure S1. Storage (circles) and loss (triangles) moduli as a function of angular frequency for the neat and Alexa-labeled Tetra-PEG slimes with *s* = 0.5 at 30 ˚C.

2. Estimation of end-modification rate

Figure S2 shows the fluorescent intensity against the molar concentrations of Alexa Fluor™ 594 NHS ester (Alexa-NHS) and Alexa-modified Tetra-PEG-GDL estimated by the fluorometer. In both panels, the intensities showed linear relationships, obeying the Beer-Lambert law. According to the left panel, the molar contribution of the Alexa to the intensity is estimated to be 3.04×10^{10} L mol^{-1}. On the other hand, from the right panel, the slope is estimated to be 1.36×10^{7} L mol^{1}. Therefore, the modification rate of the end groups in tetra-armed PEG is evaluated to be 0.0444%.

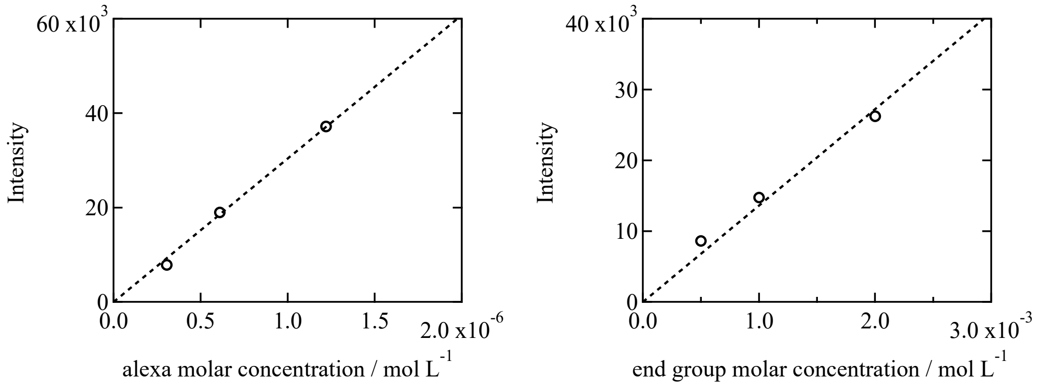

Figure S2. Fluorescent intensity as a function of Alexa-NHS concentration (left) and end group concentration of tetra-armed PEG (right).
